# Thermodynamics of ABC transporters

**DOI:** 10.1007/s13238-015-0211-z

**Published:** 2015-09-25

**Authors:** Xuejun C. Zhang, Lei Han, Yan Zhao

**Affiliations:** National Laboratory of Macromolecules, National Center of Protein Science-Beijing, Institute of Biophysics, Chinese Academy of Sciences, Beijing, 100101 China

**Keywords:** ABC transporters, differential-binding energy, energy-coupling, elastic conformational energy

## Abstract

ABC transporters form the largest of all transporter families, and their structural study has made tremendous progress over recent years. However, despite such advances, the precise mechanisms that determine the energy-coupling between ATP hydrolysis and the conformational changes following substrate binding remain to be elucidated. Here, we present our thermodynamic analysis for both ABC importers and exporters, and introduce the two new concepts of differential-binding energy and elastic conformational energy into the discussion. We hope that the structural analysis of ABC transporters will henceforth take thermodynamic aspects of transport mechanisms into account as well.

**Electronic supplementary material:**

The online version of this article (doi:10.1007/s13238-015-0211-z) contains supplementary material, which is available to authorized users.

## INTRODUCTION

### ABC transporters

Transporters are membrane proteins that typically utilize external energy to transport substrates across cellular membranes. They exist in all life kingdoms, and are commonly associated with a vast variety of cellular processes (Davidson et al., 2008). The two largest transporter families are adenosine triphosphate (ATP) binding cassette (ABC) transporters and major facilitator superfamily (MFS) transporters. Primary active transporters of the ABC family directly utilize the free energy of ATP hydrolysis to drive the substrate transport (Davidson et al., 2008), while secondary active transporters of the MFS family utilize the electrochemical potential of one substance to drive the transport of another substance (Poolman and Konings, 1993; Zhang et al., 2015). Because of the large free energy associated with ATP hydrolysis (>30 kJ/mol), ABC transporters are particularly useful for transporting substrates against high electrochemical potentials (including concentration gradients and electrostatic membrane potentials) and for unidirectional transport (Grossmann et al., 2014). It has been hypothesized that throughout evolution ABC transporters have been developed from some proton-motive force (PMF)-driven secondary active transporters (Kim et al., 2004; Venter et al., 2003). In addition, ABC transporters can be divided into importers and exporters. These two groups are believed to have separated from each other earlier on in the course of evolution before the differentiation of prokaryotes and eukaryotes (Saurin et al., 1999), with eukaryotic cells mainly possessing exporters (Bouige et al., 2002). Many human diseases can be traced back to the malfunctioning of ABC transporters (Davidson et al., 2008). Therefore, understanding the mechanisms of ABC transporter function is of significant importance.

### 3D structures of ABC transporters

A general alternating-access model was put forward for transporters nearly 50 years ago (Jardetzky, 1966). According to this model, the substrate binding site in a transporter alternates its access to the two sides of the barrier membrane, and the two corresponding conformations are referred to as inward-facing (C_In_) and outward-facing (C_Out_). An implementation of such a mechanism has been observed in 3D structures of MFS transporters, for which it was termed ‘rocker-switch’ mechanism (Huang et al., 2003). Similar conformational changes in the transport cycle have been observed for ABC transporters. For example, C_In_ and C_Out_ conformations of the maltose transporter complex MalFGK_2_ from *E. coli*, one of the best studied ABC transporter systems, have been shown to be connected through rigid-body movement between the two transmembrane (TM) domains (Chen, 2013; Khare et al., 2009).

**Figure 1:**
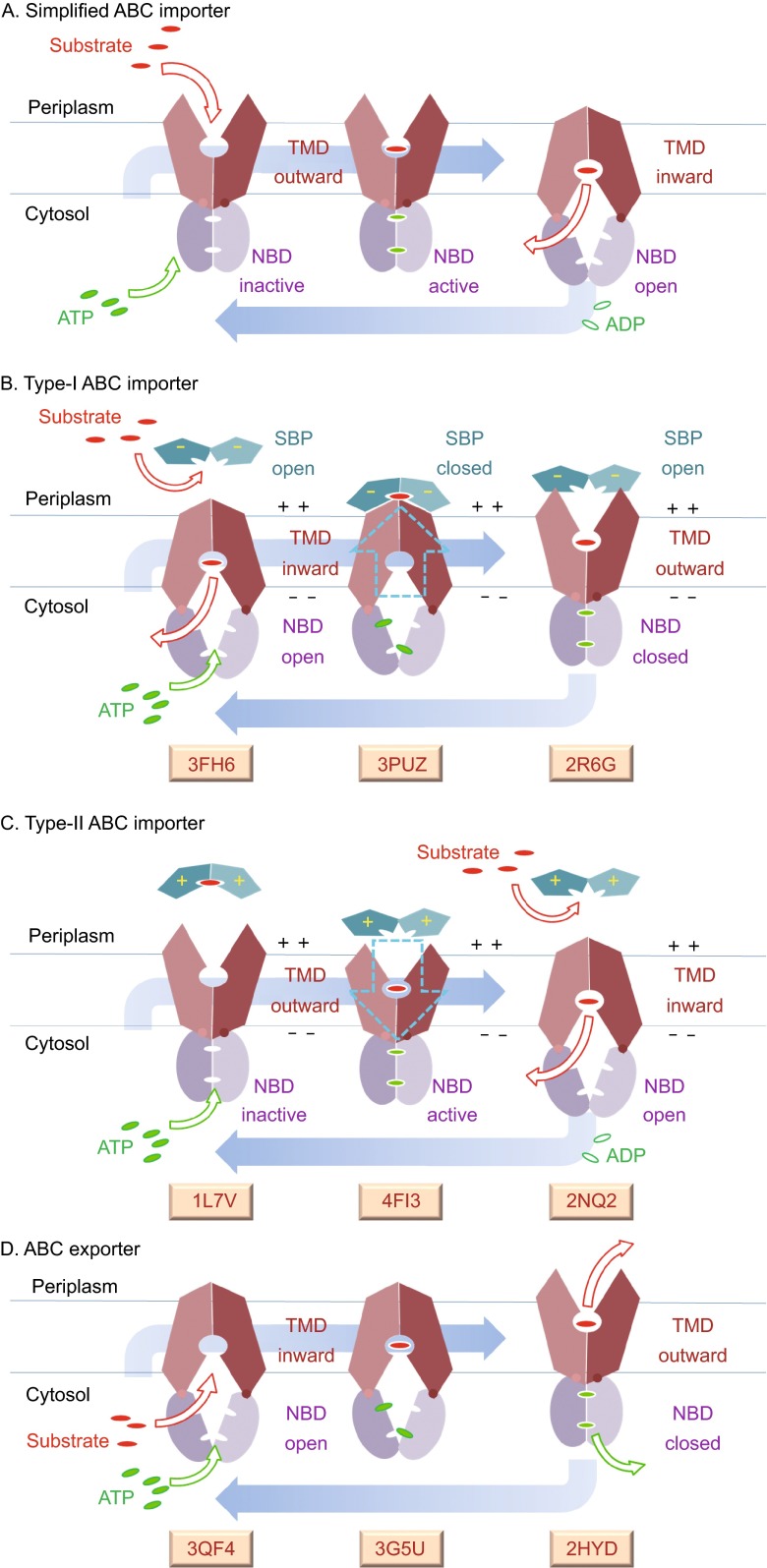
**Schematic diagrams of functional cycles of ABC transporters**. (A) Simplified ABC importer. (B) Type-I SBP-dependent importer. (C) Type-II SBP-dependent importer. In both panels (B) and (C), the cyan arrow indicates the direction of the electrostatic force applied by the membrane potential on the SBP-importer complex. This force may facilitate the formation of the ATPase active site. (D) ABC exporter. For a simple ABC exporter, the transition from the second/middle to the third/right conformation is powered by ΔG_E_. ATP hydrolysis drives the resetting of the transporter back to its resting state, C_In_. For a P-gp like exporter, the second/middle conformation is in equilibrium with the third/right one. ATP hydrolysis drives both substrate release and conformational resetting. For all panels, the first/left conformation is presumably the resting state. PDB access codes of representative crystal structures are listed at the bottom

Crystal structures have been solved for a number of ABC importers and exporters (Hollenstein et al., 2007; Kadaba et al., 2008; Kim et al., 2014; Locher et al. 2002; Pinkett et al., 2007; Reyes and Chang, 2005; Srinivasan et al. 2014). All ABC transporters contain nucleotide binding domains (NBDs) and TM domains (TMDs) (Fig. 1). The NBDs may form a head-to-tail, symmetrical homodimer (or pseudo-symmetrical heterodimer) on the cytoplasmic side of the membrane. Such an NBD dimer typically contains two ATP-binding sites in the domain interface, and it is responsible for ATP binding and hydrolysis. The TMDs usually form a parallel, symmetrical homodimer (or pseudo-symmetrical heterodimer), and the TMD dimer is involved in the formation of the path required for substrate transport across the membrane. In addition, many ABC importers require a high-affinity substrate binding protein (SBP) to deliver substrates (Rice et al., 2014). ATP hydrolysis induces a large conformational change in the NBD dimer, which is coupled to the TMD conformational change from C_Out_ to C_In_ through conserved motifs in NBD-TMD interfaces (Jones et al., 2009). Compared to importers, the TMDs of exporters usually contain longer helices protruding into the cytoplasm, where they contact NBDs (Davidson et al., 2008; Jones et al., 2009). On the one hand, available crystal structures of ABC transporters have provided a structural basis for understanding the mechanisms of coupling ATP hydrolysis in the NBDs with the conformational change of the TMDs that is associated with cross-membrane transport of the substrate. One the other hand, one should keep in mind that crystal structures are only snapshots of the structures under detergent-solubilized conditions, which are usually carefully optimized for proper crystallization, rather than obtaining the lowest-energy conformation of the protein as present *in situ* (Perez et al., 2015). Generally speaking, energy costs of conformational changes in the *in vitro* and *in vivo* situations have to be assumed to differ to some extent. Under *in vivo* conditions changes in conformation are usually accompanied by changes in membrane curvature and/or positional adjustment of the protein relative to the lipid bilayer (including its electrostatic membrane potential), whereas in a detergent-solubilized form, micelles present a different environment for the transporter.

### Current models of transport mechanisms

In the currently prevailing “switch model” of transport action (Fig. 1) (Jones et al., 2009), the NBDs form a compact dimer upon ATP binding. Through a tweezer-like movement, formation of the ATP-bound NBD dimer drives a “clothes peg”-like movement of the TMD dimer, switching from the C_In_ state to the C_Out_ state. This “clothes peg”-like movement of the TMD dimer is conceptually similar to the rocker-switch mechanism of MFS transporters. The bound ATP molecules are then hydrolyzed, resulting in (partial) dissociation of the NBD dimer and release of adenosine diphosphate (ADP) and inorganic phosphate (P_i_). In this model, binding of ATP provides the “power stroke” driving substrate transport, while ATP hydrolysis is required to reset the transport cycle to C_In_ (often referred to as a resting state). Despite an established consensus on the sequence of these conformational changes, the precise mechanisms responsible for the coupling of energy to the actual substrate transport remain elusive at best.

### Scope of this work

Many excellent reviews are now available on the structures of ABC transporters (Davidson et al., 2008; Higgins and Linton, 2004; Jones et al., 2009; Rice et al., 2014). Here, we will avoid a repeating of such structural analyses; instead, a thermodynamic description of the energy-coupling mechanisms of the ABC transporter is presented. Such a description should allow a verification of a variety of models extrapolated from experimental observations based on thermodynamic principles and formulation of some key questions on the structure-function relationship of ABC transporters. In particular, we will emphasize the importance of the two concepts of *differential-binding energy* and of *elastic conformational energy*.

## THERMODYNAMIC MODELS

The transport cycle of any transporter must follow thermodynamic laws. In particular, Gibbs free energy (ΔG) of every independent step as well as of the total isothermal-isobaric transport cycle must be negative. Generally speaking, a transporter may facilitate the substrate translocation kinetically and/or thermodynamically by (i) helping the substrate (or hydrophilic groups of the substrate) to move across the hydrophobic membrane barrier; and in addition (ii) moving the substrate against its own concentration gradient, i.e. overcoming the substrate chemical potential (Δμ(s) ≝ RT*ln*([s]_R_/[s]_L_) > 0, where the subscripts “L” and “R” stand for the states of substrate loading and releasing, respectively). In the first case, when it assumes a negative value, Δμ(s) itself may provide part of the driving force. In the second case, a substrate molecule is often captured at a high affinity site (with K_d,L_(s) << [s]_L_) on one side of the membrane and is released from a low affinity site (with K_d,R_(s) > [s]_R_) on the other side of the membrane. ABC transporters are frequently used in the second scenario.

**Figure 2:**
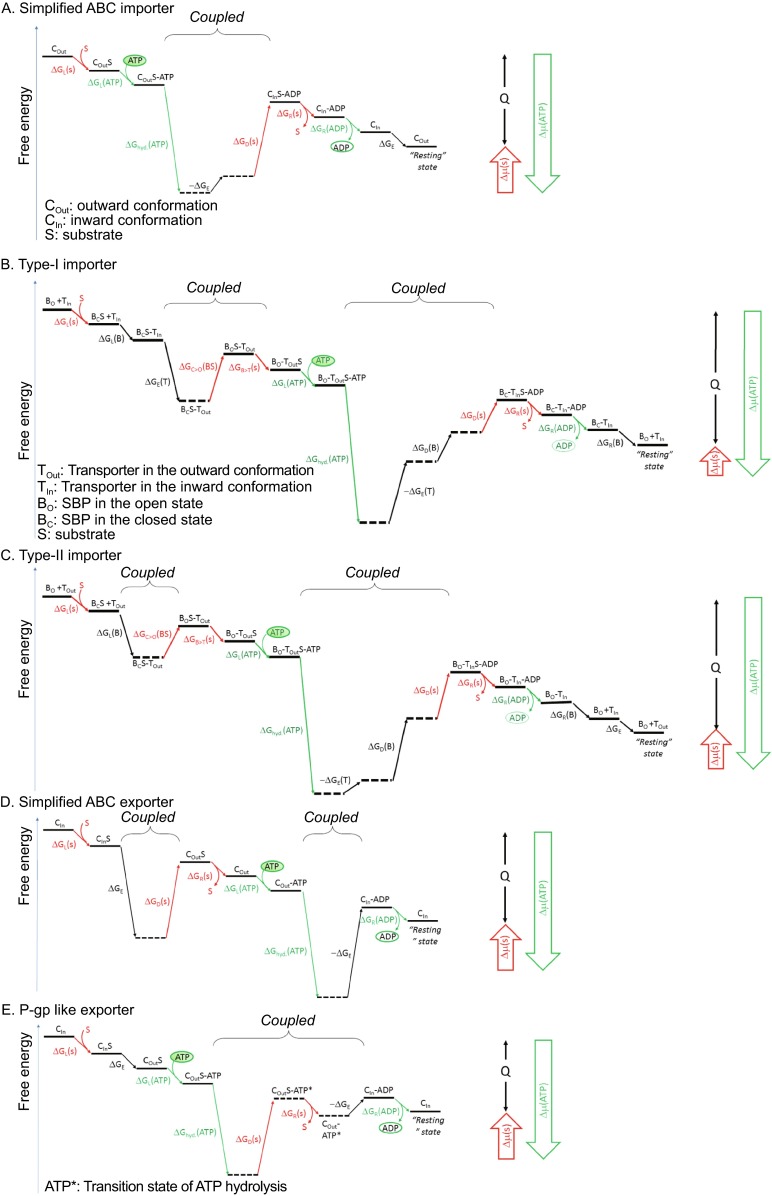
**Schematic free-energy landscape of ABC transporters**. (A) Simplified importer (See Fig. 1A). (B) Type I importer, maltose transporter complex of E. coli (MalFGK_2_-E) (See Fig. 1B and Ref. (Austermuhle et al., 2004)). (C) Type-II importer (See Fig. 1C). (D) Simplified exporter (See Fig. 1D). (E) P-gp like exporter (See Ref. (Sauna and Ambudkar, 2000)). A free-energy landscape plot describes the thermodynamic relationship between different states, without attention to kinetic issues. Horizontal lines represent states, with imaginary states in dashed lines. Tilted lines represent transitions between states (note that detailed transition-state barriers are not taken into consideration). Components in red are related to the substrate transport, those in green to ATP hydrolysis. Subscripts L, R, D, and E stand for energy terms associated with loading, releasing, differential binding, and elastics, respectively. Because the transport process is cyclical, the choice of the starting point is, in principle, arbitrary. Therefore, the starting and ending states are identical, only differing in the release of heat (Q) during one transport cycle. Thus, the end state must be below the starting state. Locally, any transition of positive ΔG must be driven by a neighboring transition of a negative ΔG. In such cases, neighboring events may be either sequentially ordered or simply clustered. For a sequential order, one event may lower the transition energy barrier of the next event, thus making the kinetics of the latter feasible. Clustered events, in contrast, occur simultaneously. Dividing such a clustered event into separate energy terms is mostly conceptual (see Appendix for further details). Note that for simplicity, release of P_i_, a product of ATP hydrolysis, is not shown. Instead, it is absorbed in the ADP terms. Nevertheless, it is entirely possible that P_i_ and ADP dissociate from the transporter at separate steps of the process

To simplify the discussion, we first focus on a model of ABC importer that contains only NBDs and TMDs. Here, it is assumed that C_In_ and C_Out_ are the only two major conformations in the transport cycle. The substrate-loading state is of the C_Out_ conformation, whereas the substrate-releasing state is of the C_In_ conformation. The two states are separated by an energy barrier, which is relevant to the kinetics of the conformational change between the two states. A free-energy landscape plot, which depicts the Gibbs free-energy terms between different states of the transport process, will be used here to facilitate the dissecting of the transport cycle. A schematic free-energy landscape plot of our simplified ABC importer is shown in Fig. 2A. Definition of the energy terms are summarized in Appendix. Key aspects of our thermodynamic scheme include (i) loading of the substrate with high affinity from the periplasmic/extracellular space; (ii) releasing of the substrate with low affinity to the cytosol; and (iii) utilizing the energy from ATP hydrolysis to drive the conformational change of the substrate-carrying transporter from the high affinity state to the low affinity state. Similar thermodynamic schemes are likely to be valid for other ATP-driven transporters, e.g. P-type ATPase importers (Bublitz et al., 2011) or energy-coupling factor (ECF) importers (Xu et al., 2013).

### Free energy associated with the substrate

Compared to an MFS transporter, an ABC transporter consumes much more energy in each transport cycle. This requirement is often related to the large sizes and/or low concentrations of the substrates, as well as to ensure directionality of the transport (Grossmann et al., 2014). High affinity of an ABC transporter towards its substrate at the loading state may facilitate transporting substrate of very low concentration. For example, if the substrate is transferred from a nanomolar environment to a micromolar environment, the associated free-energy change Δμ(s) would be RT*ln*(1000) ≈ 7RT. Such an energy increment would be difficult to be provided by the electrochemical potential of protons, for example, in a PMF-driven transporter, which generates ~6RT energy per cycle (Jiang et al., 2013). Therefore, ABC transporters become necessary in these cases.

Any component (x) that is loaded to a transport system must be released at some point during the transport cycle. Its chemical potential change (i.e. Δμ(x) ≝ RT*ln*([x]_R_/[x]_L_)) can be divided into at least three terms (Fig. S1), namely (i) the loading energy (i.e. ΔG_L_(x) ≝ RT*ln*(K_d,L_(x)/[x]_L_)); (ii) differential-binding energy (i.e. ΔG_D_(s) ≝ RT*ln*(K_d,R_(x)/K_d,L_(x))); and (iii) releasing energy (i.e. ΔG_R_(x) ≝ RT*ln*([x]_R_/K_d,R_(x))). In order for the import cycle to work sustainably, both the free-energy terms of substrate loading ΔG_L_(s) and releasing ΔG_R_(s) are usually negative. It is important to note that ΔG_D_(s), the difference in binding energy between the substrate loading site and releasing site, is a property of the transporter as well as the substrate and is independent of substrate concentrations. In case that [s]_L_ << [s]_R_, ΔG_D_(s) is necessarily of a large positive value, including not only the final positive Δμ(s) but also both -ΔG_L_(s) and -ΔG_R_(s) as well (Fig. S1). In other words, there is a significant free-energy increment (ΔG_D_(s) > 0) to be overcome during the process of converting a high-affinity site to a low-affinity site, and external energy must be provided to drive the process. In an ABC transporter, a significant portion of the free energy of ATP hydrolysis is often utilized to overcome this positive ΔG_D_(s). As a simplified model, we assume here that the differential-binding energy, ΔG_D_(s), is associated with the major substrate-carrying conformational change, e.g. C_Out_-to-C_In_ for an importer. However, alternative mechanisms are possible, which we will discuss below.

### Free energy associated with ATP

ATP molecules are considered to contain high chemical energy, and their hydrolysis to ADP and P_i_ generates more than 30 kJ/mol (~12RT) energy. Currently, it is still not entirely clear whether one or two ATP molecules are hydrolyzed in a typical transport cycle. Part of the reason for this uncertainty is the presence of basal ATPase activity of ABC transporters in the absence of substrates, especially in *in vitro* assays (Woo et al., 2012). In the ATP-dependent anion channel CFTR it has been shown that only one ATP molecule is hydrolyzed by its asymmetric NBD heterodimer per functional cycle (Gadsby et al., 2006). In addition, in human MRP1 (a fused TMD_1_-NBD_1_-TMD_2_-NBD_2_ ABC transporter), ATP hydrolysis predominantly occurs in the second NBD (Zhang et al., 2003). In comparison, in a so-called alternating catalytic scheme (Senior et al., 1995), the two ATP-binding sites in the NBD dimer take turns in providing energy for the transporter, with the result that probably only one ATP molecule is consumed per transport cycle. In contrast, based on data from a vanadate-trapped transition-state intermediate, it was proposed that P-glycoprotein (P-gp) consumes two ATP molecules per transport cycle (Sauna and Ambudkar, 2000). In this model, one ATP molecule was used for ejecting the substrate at the transition state and the other one for resetting the transporter back to the resting state following the transition state. However, the presence of vanadate drastically changes the energy landscape of the ‘transition state’, converting the latter from a high-energy, unstable state (by definition) to a low-energy, stable state. Thus, it is feasible that in the absence of vanadate, the resetting step is in fact powered by the P_i_ release (which was substituted by vanadate in the reported experiment) from the ‘first’ ATP hydrolysis. Therefore, we consider hereafter that only one ATP molecule is consumed per transport cycle for a P-gp like transporter. Since both ATP loading and ADP release are referred to the same intracellular pool, the change in Gibbs free-energy of an ATP molecule during a transport cycle is exactly the same as that of ATP hydrolysis in the cytoplasm. The latter is defined as Δμ(ATP) ≝ RT*ln*(([ADP]•[P_i_])/([ATP]•K_eq.,W_)), where the subscript “W” indicates a reaction in water. This free-energy value of ATP hydrolysis is determined by the cellular contents (i.e. [ATP], [ADP], and [P_i_]), and it sets the upper limit how much energy may be gained from ATP hydrolysis in a transport cycle. In aquatic solution, K_eq.,W_ is in the order of 1 × 10^7^ mol/L (i.e. (55.6 mol/L) exp((30 kJ/mol)/RT), where 55.6 mol/L is the molar concentration of water). Such a large K_eq.,W_ strongly favors ATP hydrolysis. Moreover, the intracellular ATP concentration is about 1–10 mmol/L, and that of ADP is usually lower. Taken together, an ABC transporter has the ability to utilize much more energy per transport cycle than a secondary active transporter, allowing the transport of more energy-demanding substrates. However, not all of the free energy of ATP hydrolysis (Δμ(ATP)) is directly used to drive the C_Out_-to-C_In_ conformational change of the transporter, or to overcome the energy increment associated with the differential-binding energy of the substrate (ΔG_D_(s) > 0). Some portion of Δμ(ATP) must be utilized for ATP loading as well as ADP releasing. For instance, the ATP-loading free energy is denoted as ΔG_L_(ATP) ≝ RT*ln*(K_d,Out_(ATP)/[ATP]), where the subscript “Out” stands for the C_Out_ conformation. The free-energy change of ATP hydrolysis within the transporter is ΔG_hyd._(ATP) (see Appendix):

$$\Delta {\text{G}}_{{{\text{hyd}}.}} \left( {{\text{ATP}}} \right)\mathop {\text{ = }}\limits^{{{\text{def}}}} {\text{RT}}ln\left( {\left( {{\text{K}}_{{{\text{d,In}}}} \left( {{\text{ADP}}} \right)\cdot{\text{K}}_{{{\text{d,In}}}} \left( {{\text{P}}_{{\text{i}}} } \right)} \right)/\left( {{\text{K}}_{{{\text{d,Out}}}} \left( {{\text{ATP}}} \right)\cdot{\text{K}}_{{{\text{eq}}.,{\text{W}}}} } \right)} \right)$$

In importers, it is this ΔG_hyd._ term that drives the C_Out_-to-C_In_ conformational change, during which the positive ΔG_D_(s) is likely to be compensated for. It is noteworthy that the more negative ΔG_L_(ATP) is (by decreasing K_d,Out_(ATP)), the closer to zero ΔG_hyd._ becomes. In other words, a stronger ‘power-stroke’ associated with ATP binding is achieved at the expense of reducing the hydrolysis power of the bound ATP.

### Simplified importer model

For our simplified ABC importer (Fig. 1A), ATP loading is coupled with substrate binding, and ATP hydrolysis is directly coupled to substrate import. Thus, in the absence of a substrate in the C_Out_ conformation, the NBD dimer is most likely to be ATPase-inactive, in order to prevent futile ATP hydrolysis. Substrate binding in C_Out_ promotes completion of the ATPase catalytic center and triggers the subsequent C_Out_-to-C_In_ conformational change. Part of the ATP hydrolysis energy is likely to be stored in the C_In_ conformation as elastic conformational energy, ΔG_E_ (<0) (Fig. 2A). In general, any reversible conformational change of higher free-energy can be utilized for storage of ΔG_E_. As an analogy, a membrane protein can be thought as a boat floating in water. Loading cargos will push the boat deeper into the water, thus storing some energy as ΔG_E_. In case the cargos are thrown into the water, the boat will be pushed up again by the released ΔG_E._ The elastic conformational energy of the transporter should be sufficient thermodynamically for driving the C_In_-to-C_Out_ conformational change, thus completing the transport cycle. However, there may be an energy barrier between the two conformations that determines the kinetics of the substrate-free C_In_-to-C_Out_ transition. Therefore, releasing of the elastic conformational energy is likely to be a controlled event. In this simple ABC importer model, substrate releasing lowers this energy barrier, resulting in the C_In_-to-C_Out_ conformational change. Thus, the resting state of such an importer model is likely to be C_Out_ rather than C_In_.

### Simplified exporter model

A similar model can be proposed for an ABC exporter (Figs. 1D and 2D). In such a simplified exporter model, ATP hydrolysis is coupled with substrate dissociation and results in storage of the elastic conformational energy ΔG_E_ in the C_In_ conformation instead of being directly utilized in substrate transport. Thus ΔG_E_ of such an exporter is likely to be substantially larger than that of the simple importer, large enough to overcome the positive differential-binding energy of the substrate, ΔG_D_(s). Accordingly, substrate binding triggers release of the stored ΔG_E_ and promotes the substrate-carrying C_In_-to-C_Out_ conformational change. Further, the ATP hydrolysis should be coupled with releasing substrate from the extracellular or periplasmic side in the C_Out_ state, followed by the substrate-free C_Out_-to-C_In_ conformational change. Otherwise, backward transport of the substrate might occur, and the free energy of ATP hydrolysis would become futile. Therefore, the energy-coupling mechanism of the simple ABC exporter appears drastically different from that of the simple ABC importer, for example in terms of usage of ΔG_E_. This proposition appears to be consistent with the overall differences between ABC importers and exporters observed in 3D structures as well as in phylogenetic analyses (Jones et al., 2009).

### Model of P-gp like exporter

In both simplified importer and exporter models discussed above, we made the assumption that ΔG_D_(s) is associated with the major conformational change occurring while substrate is carried across. For exporters, such an assumption would require a significant amount of elastic conformational energy, ΔG_E_, to be stored in the C_In_ conformation to allow ΔG_D_(s) to be compensated during the C_In_-to-C_Out_ conformational change. Although such an exporter model is feasible, alternative energy-coupling mechanisms have been implicated previously for some actual exporters. For instance, when ΔG_E_ is small, a strong ΔG_hyd._(ATP) may be coupled directly to the release of the substrate (Fig. 2E) (Sauna and Ambudkar, 2000). In this case, the affinity towards the substrate remains essentially unchanged between C_In_ and C_Out_ (before ATP hydrolysis), alleviating the potentially burdening requirement of a large negative ΔG_E_. Instead, the large ΔG_D_(s) obligational for the unidirectional transport is associated with a small yet powerful conformational change during ATP hydrolysis. Such an energetic conformational change can, however, be trapped experimentally by using an ADP-vanadate complex. The corresponding trapped ‘transition state’ shows a more than 30-fold lower substrate affinity when compared to the ground state (Sauna and Ambudkar, 2000). Therefore, for this type of ABC exporters, it is the ATP hydrolysis, instead of the elastic conformational energy, that directly powers the ejection of the substrate (i.e. compensating the large ΔG_D_(s)). In addition, if ΔG_E_ is small, the substrate-carrying C_In_ and C_Out_ states may readily equilibrate. This may provide a partial explanation to the previous observation that the ABC exporter, transporter associated with antigen processing (TAP), becomes a bidirectional, yet low-efficient transporter when mutations occur in its NBD dimer (Grossmann et al., 2014). Furthermore, the lipopolysaccharide (LPS) transporter of Gram-negative bacterial, LptB_2_FG, extracts its substrate LPS from the periplasmic face of the inner membrane (IM) and transport LPS to the outer leaflet of OM, through a bridge protein (LptC) spanning across the periplasm and a trans-OM channel (LptDE) (Qiao et al., 2014; Sherman et al., 2014). The ABC exporter LptB_2_FG is the sole energy source for the entire process of LPS transport. Interestingly, loading of the substrate LPS occurs in the C_Out_ instead of C_In_ conformation. Since a transition state by definition cannot be an energy-generating state, the ejection of the substrate into the downstream carrier LptC most likely occurs at the post-transition state of the ATP hydrolysis. Interestingly, a recent report on the flippase PglK from *Campylobacter jejuni* suggests a similar mechanism of transporting lipid-linked oligosaccharide from the inner leaflet to the outer leaflet of the periplasm membrane (Perez et al., 2015).

In reality, some ABC exporters might utilize a hybrid mechanism of the two types of ABC exporters, whereby compensation of ΔG_D_(s) is shared by both ΔG_E_ and ΔG_hyd._(ATP). Furthermore, for a given ABC exporter, the distribution of ΔG_D_(s) in both ΔG_E_ and ΔG_hyd._(ATP) may vary from substrate to substrate, because of their differences in *K*_d_(s) values in the conformations of C_In_, C_Out_, and the post-transition state of ATP-hydrolysis.

## STRUCTURAL BASIS OF THE ENERGY-COUPLING MECHANISM

A goal of structural studies of ABC transporters is to understand the structural basis of the above discussed thermodynamic principles. Since the C_In_ conformation stores some form of elastic conformational energy (ΔG_E_) in both importers and exporters, an important question to be addressed is as to ‘what exactly the nature of such energy is?’ Because the TMDs of ABC transporters are highly variable in terms of their primary structures (Davidson et al., 2008), it is likely that the energy-storage/coupling mechanisms differ somewhat for the different types of ABC transporters.

### Effects of membrane potential

Hypothetically, an ABC transporter may use a change of electrostatic charge to stabilize a particular state. In general, in the presence of a membrane potential, a membrane protein may change its conformational state upon changing its electrostatic charges, thus re-balancing the electrostatic force with hydrophobic mismatch forces (Phillips et al., 2009; Zhang et al., 2014). For ABC transporters, such a charge-induced conformational change may function as a trigger for releasing ΔG_E_, by lowering the transition-state energy barrier from C_In_ to C_Out_.

As a putative mechanism (Fig. S2), in the absence of a substrate, the C_In_ state of an ABC exporter may carry a high p*K*_a_ protonation site and become protonated. Through interaction with the negative-inside membrane potential, this extra positive charge from the proton is subjected to an inward force (i.e. towards to the cytosolic side) and thus may stabilize the C_In_ conformation. Upon substrate binding, the proton-titratable site may be disrupted and become deprotonated. Thus the C_In_ conformation of the exporter becomes less stable and the stored ΔG_E_ is released to drive the subsequent C_In_-to-C_Out_ conformational change. In agreement with this argument, in human MRP1, mutations of D1084N/A/V/R at the solvent-inaccessible, conserved acidic-residue position 1084 in TM14 resulted in a loss of transport activity for all drugs tested; yet the mutation variants showed increased binding affinity by 2–3 folds for the substrates (Zhang et al., 2003). In addition, the 1084E variant maintained ~50% of WT activity. In contrast, many mutations at other positions in TM14 exhibited reduced substrate-binding affinities. These observations suggest that the electrostatic interaction between protonated D1084 and membrane potential stabilizes the substrate-free transporter in the C_In_ state and plays critical roles in both substrate sensing and transport. Since the proton is not required to be transported across the membrane, there may not be proton consumption during the transport cycle. Such a hypothetical mechanism can be verified experimentally by testing the dependence of ABC exporters on pH as well as the membrane potential. In support of such a hypothesis, it has been reported that when mutated in the NBD, some ABC exporters function as PMF-driven transporters (Davidson et al., 2008). For example, the isolated TMD of multidrug-resistance ABC exporter LmrA of *Lactococcus lactis* was shown to mediate pH-dependent transport (Venter et al. 2003), suggesting that the TMD dimer contains pH-sensing elements. LmrA and P-gp are shown to share a conserved acidic residue in TM6 at the substrate entrance from the cytosol side (Shilling et al., 2005). In addition, ATP-dependent, multidrug-efflux transporters such as P-gp and BCRP are shown to export substrates more efficiently at lower pH values (Li et al., 2011; Varma et al., 2005). If the hypothesized evolutionary relationship between ABC transporters and PMF-driven secondary active transporters (Kim et al., 2004) is correct, then the proton-sensing function may still be available to some ABC transporters. Such a regulatory mechanism of ABC transporters may provide a common approach to solving the substrate-selectivity problems of multidrug transporters dealing with substrates of a wide range of structural and chemical diversity. Similar protonation-mediated, regulatory mechanisms of conformational statuses have also been proposed for MFS transporters (Heng et al. 2015) as well as GPCR signaling proteins (Zhang et al., 2014).

Alternatively, in the presence of a membrane potential, negatively charged substrates *per se* generate an outward force, which may trigger the C_In_-to-C_Out_ conformational change of the exporter. Indeed, for effective transport, MRP1-like ABC exporters often require a negatively charged co-factor, such as glutathione (GSH) (Wesolowska, 2011). For example, good MRP1 substrates contain up to four negatively charged chemical groups such as a carboxylic acid group, sulfonic acid group or sulfate group (Seelig et al., 2000). In addition, substrates can either be conjugated to GSH by special enzymes or simply co-transported with GSH (Deeley et al., 2006; Zhang et al., 2003). As shown recently in the crystal structure of yeast Amt1, a binding site for the GSH moiety is located near the substrate entrance in the C_In_ conformation (PDB ID: 4MYH) (Srinivasan et al., 2014).

### Two types of ABC importers

Nearly all bacterial ABC importers utilize SBPs to deliver ligands (Fig. 1B and 1C). Association and dissociation of an SBP with its cognate transporter can be considered as part of the conformational change of the transporter. Thus, from the energy-coupling point of view, the free energy associated with loading (ΔG_L_) and releasing (ΔG_R_) of SBP can be considered as part of the elastic conformational energy and is provided by the ATP hydrolysis in the form of differential binding energy of the SBP (i.e. ΔG_D_(B) in Fig. 2B and 2C).

On the base of the size of their TMDs, SBP-dependent ABC importers can be divided into two classes (Rice et al., 2014). Each type I importer typically contains 5 × 2 TM helices. The following properties are attributed to type I importers (Fig. 1B): (i) the alternating access mechanism is implemented as rigid-body movement; (ii) their resting state is the nucleotide-free C_In_ conformation; (iii) in the absence of an SBP, a type I ABC importer shows negligible ATPase activity; (iv) binding of the SBP, but not free substrate, stimulates the ATPase activity in both micelles and proteoliposomes (Davidson et al., 1992; Rice et al., 2014). The *E. coli* maltose transporter complex, MalFGK_2_-E, is a representative of type I importers, and its thermodynamic free-energy scheme is shown in Fig. 2B. A major feature of the free-energy plot of this SBP-dependent ABC importer is its large ΔG_E_. This elastic conformational energy should be large enough to compensate the differential-binding energy of the substrate during the translocation from SBP (i.e. maltose binding protein (MBD), or MalE) to TMD, concomitant with the C_In_-to-C_Out_ conformational change. For the maltose transport system, the *K*_d_ of SBP towards maltose is 0.1–1 μmol/L, while the *K*_M_ of the TMD is about ~1 mmol/L (Covitz et al., 1994). Thus the differential-binding energy is estimated to be in the order of RT*ln*(1000) (or ~7RT), so may the elastic conformational energy ΔG_E_ be. Structurally, reduction of the binding affinity between the substrate (maltose) and SBP (MBD/MalE) is achieved through the protrusion of a so-called scoop-loop from the TMD into the maltose binding site in SBP (PDB ID codes: 4KHZ and 4KI0) (Oldham et al., 2013), and the energy required for this structural change is provided by the SBP-TMD binding energy, which may be considered as part of ΔG_E_ as mentioned above. Intriguingly, for mutants that are not stable in the C_In_ state (e.g. by lowering the transition-state energy barrier), i.e. variants that are unable to store ΔG_E_ in C_In_, the maltose transporter becomes an SBP-independent, constitutively active ATPase (Covitz et al., 1994; Davidson et al., 1996; Davidson et al., 1992). It suggests that part of the functions of the SBP associated with the type I importer is to overcome the transition-state energy barrier from C_In_ to C_Out_.

Interestingly, for all three SBPs of the type I importers that have been well-studied structurally, the values of isoelectric point (pI) are below 6.0. In particular, the pI of MalE of the *E. coli* MalFGK_2_-E complex is 5.5 (Oldham et al., 2013); 5.1 for MetQ of the *E. coli* MetN_2_I_2_-Q complex (Kadaba et al., 2008); and 5.6 for ModA of *Archaeoglobus fulgidus* ModB_2_C_2_-A complex (Hollenstein et al., 2007). In addition, based on their gene operons, the SBPs of PotD/F and OppA are predicted to associate with type I importers (Rice et al., 2014). These SBPs also have acidic pI values, namely 5.2 for *E. coli* PotD; 6.4 for *T. pallidum* PotD; 5.9 for *E. coli* PotF; 6.1 for *S. typhimurium* OppA; and 5.8 for *Y. pestis* OppA (Berntsson et al. 2010). This observation raises the question as to whether such a property of SBPs of the type I ABC importers is partially responsible for triggering the release of ΔG_E_? Since the presence of a membrane potential is not absolutely required for ATPase activity, at least under some *in vitro* conditions (Orelle et al., 2008), the binding of SBP *per se* appears to be able to induce the C_In_-to-C_Out_ conformational change. It indicates that SBP-TMD interaction is thermodynamically favored in the C_Out_ state over that in the C_In_ state. However, in the presence of a negative-inside membrane potential, binding of an acidic SBP in the C_In_ state may have another advantage: it triggers an outward movement of the SBP-importer complex towards the periplasmic side (see Fig. 1B). Such a movement may in turn facilitate the C_In_-to-C_Out_ conformational change of the TMD and concomitant substrate loading from the SBP to TMD. Therefore, the electrostatic energy as well as the binding energy of the SBP-TMD complex may be considered as a form of *in vivo* ΔG_E_ that drives substrate loading. In support of such a hypothesis, a complex crystal structure of substrate-bound MalE (SBP) with the TMD of the MalFGK_2_-E system has been captured in a conformation containing occluded TMD, an intermediate state between C_In_ and C_Out_ (PDB ID: 3PV0) (Oldham and Chen, 2011). It is conceivable that in the presence of a membrane potential, the observed SBP-induced conformational change would proceed to the C_Out_ state.

Moreover, a typical type II ABC importer contains 2 × 10 TM helices (Rice et al., 2014). A paradigm of type II importers is the vitamin B_12_-transporter, BtuCD-F, of *E. coli* (Korkhov et al., 2012; Korkhov et al., 2014). The following properties are attributed to this type of importers (Figs. 1C and 2C): (i) The alternating access mechanism is based on a rearrangement of TM helices in these importers. (ii) The resting state is thought to be an ATPase-inactive C_Out_ state. (iii) Substrate-loading from the SBP to the TMD dimer appears to enhance the affinity between the SBP and TMD (Rice et al., 2014). In contrast to type I importers, some SBPs of type II importers are found to have basic pI. For examples, BtuF of *E. coli* has a pI of 8.8, and FbpA of *Pasteurella haemolytica* 8.2 (Berntsson et al., 2010). Because of the negative-inside membrane potential, binding of a basic SBP favors an inward movement of the TMD, which may trigger a subtle conformational change of the latter. In this way, the substrate loading on the periplasmic side may trigger the formation of the ATPase catalytic site in the cytosolic NBD dimer, and subsequently cause a C_Out_-to-C_In_ conformational change (Fig. 1C). Interestingly, the ATP-bound state of type II importer BtuC_2_D_2_-F of *E. coli* is captured in an SBP-bound complex crystal structure of an occluded TMD conformation (PDB ID: 4FI3) (Korkhov et al., 2012). This structural observation suggests that the occluded state of the TMD that is induced by binding of a basic SBP may couple the substrate-loading to the formation of the ATPase catalytic site.

Although the size-based categorization of SBP-dependent ABC importers has its merits, the correlation between the size of their TMD and mechanisms responsible for energy coupling may not be as strong as assumed, and should be based on statistically sound structural and functional data. Bioinformatics analysis of the *E. coli* genome has identified 44 putative SBP-depended ABC importers (Linton and Higgins, 1998). Using the Uniprot database (http://www.uniprot.org), we identified the number of TM helices present in TMD dimers, together with the pI values of the SBPs in 35 importers (Table S1). Among the 35 ABC importer complexes, 22 complexes display pI values in their SBPs that are below 6.5, with an average pI of 5.7. In addition, 9 of the complexes possess pI values >7.8, with an average pI of 8.3. Nevertheless, there appears to be no clear correlation between the sizes of the TMDs dimer and the pI values of the SBPs. This intriguing finding suggests the possibility that ABC importers may be categorized in an alternative manner that is based on the pI values of their SBPs. The two groups of importers that have acidic and basic SBPs, respectively, may use distinct mechanisms to stimulate ATPase activity.

## CONCLUDING REMARKS

A hallmark of nearly all ABC transporters is that substrate loading triggers ATP hydrolysis, which in turn results in the C_Out_-to-C_In_ conformational change. For importers, this major conformational change directly results in substrate transport from C_Out_ to C_In_. For exporters, the substrate is likely to be ejected into the extracellular space during the C_Out_-to-C_In_ conformational change. In both importers and exporters some part of the ATP hydrolysis energy is probably stored as elastic conformational energy, to be used to drive the latter steps of the transport process. Therefore, sensing substrate binding is essential for all types of ABC transporters. Apart from what discussed above, it is entirely possible that other mechanisms may also have been employed by evolution to function as sensors for substrate binding. Thus, many of the key questions about substrate sensing remain to be addressed experimentally before we will fully understand the mechanisms of ABC transporters. It is our hope that the arguments presented here add a more thermodynamic angle to the on-going discussions in the ABC transporter research field.

## Electronic supplementary material

Supplementary material 1 (PDF 630 kb)

## ABBREVIATIONS

ABC: ATP binding cassette (transporters)
ATP: adenosine triphosphate
C_In_: inward-facing conformation
C_Out_: outward-facing conformation
NBD: nucleotide binding domain
SBP: substrate binding protein
TMD: transmembrane domain
