# Supplementary material for: Thermodynamics of ABC transporters
Source: Protein Cell. 2015 Sep 25;7(1):17–27. doi: 10.1007/s13238-015-0211-z (PMC4707154; doi:10.1007/s13238-015-0211-z)
Supplement: Supplementary file 1 — Supplementary material 1 (PDF 630 kb) [file 13238_2015_211_MOESM1_ESM.pdf]

Thermodynamics of ABC Transporters

Supplementary Information

Authors: Xuejun C. Zhang*, Lei Han, and Yan Zhao

Figures

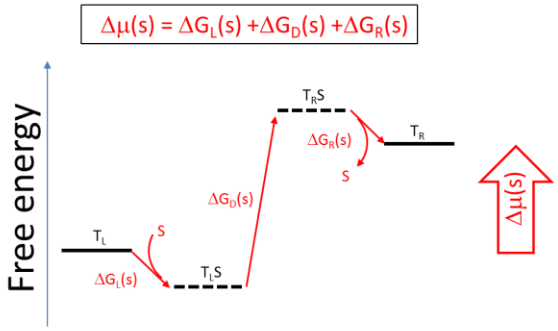

Figure S1. Differential binding energy.

Relationship between chemical potential and differential binding energy is depicted in a free-energy plot. T and S stand for the transporter and substrate, respectively. Subscripts L and R stand for loading and releasing states, respectively. Since $\Delta\mu(s) > 0$, the process shown here would not occur spontaneously and must be driven by external energy.

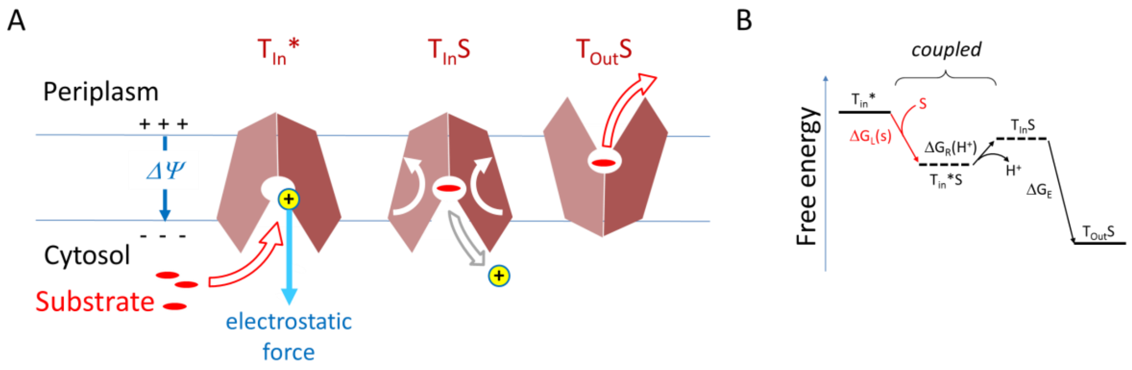

Figure S2. Putative mechanism of electrostatic interaction in ABC exporter.

A. Schematic diagram of an ABC exporter in the process of substrate binding-triggered C_{In}-to-C_{Out} conformational change. Only TMDs are shown. Protonated states are labeled with asterisk (*). The electrostatic force applied to the transporter is shown as a cyan arrow, which is balanced by the hydrophobic mismatch force. Withdrawing of this electrostatic force upon substrate binding-induced deprotonation results in a rotation torque (white arrows) in the TMDs and release of ΔG_E. B. Free energy plot. $\Delta G_R(H^+)$ is the free-energy of deprotonation. Protonation/deprotonation can be considered as part of the conformational change of the transporter complex, if the proton is not consumed during the transport cycle. Alternatively, $\Delta G_R(H^+)$ can be considered as the activation energy required to overcome the transition-state energy barrier at $T_{In}S$.

Tables

Table S1. List of SBP-dependent ABC importers from *E. coli*

TMDs	TM #	SBPs	pI	TMDs	TM #	SBPs	pI
YaeE (MetI)	10	YaeC (MetQ)	5.1	PhnE	12	PhnD	8.5
AraH	20	AraF	6.2	PotB, PotC	12	PotD	5.2
ArtM, ArtQ	10	ArtI	5.8	PotH, PotI	12	PotF	5.8
BtuC	20	BtuF	8.8	ProW	12	ProX	5.9
CysU, CysW	13	CysP	7.8	PstA, PstC	12	PstS	8.4
DppB, DppC	13	DppA	6.2	RbsC	12	RbsB	6.9
FecC, FecD	17	FecB	8.8	SapB, SapC	12	SapA	6.9
FepG, FepD	18	FepB	5.8	SfuB	12	SfuA	6.8
FhuB	17	FhuD	6.0	TauC	14	TauA	7.8
GlnP	10	GlnH	8.4	UgpA, UgpE	12	UgpB	6.0
GltJ, GltK	11	YbeJ	8.6	XylH	22	XylF	5.0
HisM, HisQ	10	HisJ	5.5	YehW, YehY	17	YehZ	5.8
LivM, LivH	19	LivK	5.5	YejB, YejE	13	YejA	6.0
MalG, MalF	14	MalE	5.5	YhdX, YhdY	16	YhdW	5.2
MglC	16	MglB	5.7	YjcV	20	YjcX	6.5
ModB	10	ModA	7.8	YrbE	10	YrbD	4.8
NikB, NikC	12	NikA	5.8	YtfT, TjfF	19	YtfQ	6.7
OppB, OppC	12	OppA	6.1				

Appendix

Free energy terms associated with an ABC transporter

(1) Total free energy

$$\Delta\mu(s) + \Delta\mu(ATP) = -Q < 0$$

(Second law of thermodynamics)

where Q is the heat released in one transport cycle; and $\Delta\mu(ATP)$ is the free energy of ATP hydrolysis.

$$\Delta\mu(ATP) \stackrel{def}{=} RT\,ln(([ADP]\bullet[P_i])/(K_{eq.,W}\bullet[ATP])) \approx -30 \text{ kJ/mol}$$

$$K_{eq.,W} \stackrel{def}{=} [ADP]_{eq.}\bullet[P_i]_{eq.}/[ATP]_{eq.}$$

where $K_{eq.,W}$ is the equilibrium constant of ATP hydrolysis reaction in water (or cytoplasm).

(2) Free energy associated with substrate loading/releasing

$$\Delta\mu(s) = \Delta G_L(s) + \Delta G_D(s) + \Delta G_R(s)$$

where

$$\Delta\mu(s) \stackrel{def}{=} RT\,ln([s]_R/[s]_L) > 0$$

(free energy associated with substrate transport)

$$\Delta G_L(s) \stackrel{def}{=} RT\,ln(K_{d,L}(s)/[s]_L) < 0$$

(free energy of substrate loading, *e.g.* in C_{Out} for an importer)

$$\Delta G_D(s) \stackrel{def}{=} RT\,ln(K_{d,R}(s)/K_{d,L}(s)) > 0$$

(differential-binding energy)

$$\Delta G_R(s) \stackrel{def}{=} RT\,ln([s]_R/K_{d,R}(s)) < 0$$

(free energy of substrate releasing, *e.g.* in C_{In} for an importer)

(3) Free energy associated with ATP/ADP loading, releasing, and hydrolysis

$$\Delta\mu(ATP) = \Delta G_L(ATP) + \Delta G_{hyd.}(ATP) + \Delta G_R(ADP)$$

where

$\Delta G_L(ATP) \stackrel{\text{def}}{=} RT\,ln(K_{d,Out}(ATP)/[ATP]) < 0$

(binding energy of ATP in C_{Out})

$\Delta G_R(ADP) \stackrel{\text{def}}{=} RT(ln([ADP]/K_{d,In}(ADP)) + ln([P_i]/K_{d,In}(P_i))) < 0$

(releasing energy of ADP from C_{In})

$\Delta G_{hyd.}(ATP) \stackrel{\text{def}}{=} RT\,ln(K_{eq.,T}/K_{eq.,W}) < 0$

(free energy of ATP hydrolysis inside the transporter)

$K_{eq.,T} \stackrel{\text{def}}{=} (K_{d,In}(ADP) \bullet K_{d,In}(P_i))/K_{d,Out}(ATP)$

(referred to as the equilibrium constant of the ATP hydrolysis reaction in the transporter)
